# Tin Oxide-Carbon-Coated Sepiolite Nanofibers with Enhanced Lithium-Ion Storage Property

**DOI:** 10.1186/s11671-017-1979-y

**Published:** 2017-03-23

**Authors:** Kai Hou, Xin Wen, Peng Yan, Aidong Tang, Huaming Yang

**Affiliations:** 1 Centre for Mineral Materials, School of Minerals Processing and Bioengineering, Central South University, Changsha, 410083 China; 2 Hunan Key Lab of Mineral Materials and Application, Central South University, Changsha, 410083 China; 3 School of Chemistry and Chemical Engineering, Central South University, Changsha, 410083 China; 4 State Key Lab of Powder Metallurgy, Central South University, Changsha, 410083 China

**Keywords:** Sepiolite nanofibers, Tin oxide-carbon decoration, Cycling performances, Lithium-ion storage property

## Abstract

Natural sepiolite (Sep) nanofibers were coated with carbon and nanoscale SnO_2_ to prepare an emerging nanocomposite (SnO_2_–C@Sep), which exhibited enhanced electrochemical performance. Sepiolite could act as a steady skeleton, carbon coating principally led sepiolite from an isolated to an electric state, and decoration of nanoscale SnO_2_ was beneficial to the functionization of sepiolite. Cycling performances indicated that SnO_2_–C@Sep showed higher discharge capacities than commercial SnO_2_ after 50 cycles. The nanocomposite SnO_2_–C@Sep possessed enhanced lithium storage properties with stable capacity retention and low cost, which could open up a new strategy to synthesize a variety of functional hybrid materials based on the cheap and abundant clay and commercialization of lithium-metal oxide batteries.

## Background

Rechargeable lithium-ion batteries (LIBs) have received an interesting attention for their high energy density, high voltage, stable cycling, and environment-friendly properties. After the first announcement of commercialization of tin dioxide (SnO_2_) as a negative electrode of lithium-ion batteries, this transition metal oxide anode has received much concern. There are still two main factors that hinder the development of SnO_2_ for lithium secondary batteries: First, the de-/lithiation mechanism of SnO_2_ could be described by a two-step reaction, the conversion reaction (SnO_2_ + 4Li^+^ + 4e^−^ → Sn + 2Li_2_O) with the theoretical capacity of 712 mAh g^−1^ and the alloying reaction (Sn + xLi^+^ + xe^−^ ↔ Li_x_Sn, 0 ≤ *x* ≤ 4.4) with the theoretical capacity of 782 mAh g^−1^ [1, 2]. Accordingly, SnO_2_ as an electrode material shows large initial irreversible capacity and low initial coulombic efficiency (ICE), about 52.4% of the theoretical ICE value if conversion reaction is fully irreversible; that is more than twice of commercial graphite (372 mAh g^−1^) [3]. Second, SnO_2_ anode has still not been achieved mainly due to its capacity to rapidly fade during cycling, huge volume changes of up to 300%, and the severe interparticle aggregation of SnO_2_, which usually results in the loss of electrical contact with current collector caused by large volume changes and pulverization occurring during lithium insertion/extraction [4]. Various methods have been developed to overcome the abovementioned problems such as morphology and size control of SnO_2_ nanoparticles [5–12] and SnO_2_-based composite materials [4, 13–21]. The multi-component materials, such as carbon nanotubes (CNTs) or graphene, have been employed to improve the conductivity and mechanical strength, as well as to buffer volume changes though with a complex preparation process and higher cost of C-based materials.

To date, many kinds of minerals have been studied for the preparation of advanced materials [22–31]; the recent research on anode material with natural mineral sepiolite (Sep) has also been carried out. Sep is a hydrated magnesium clay mineral of fibrous morphology with Si_12_O_30_Mg_8_(OH,F)_4_(H_2_O)_4_·8H_2_O as the unit cell formula. Apart from the fibrous macroscopic and high specific surface, rich reserves and low cost are also well known [32–34]. On the basis of the above features, Sep has been paid much more attention in the world and many have started to attempt using Sep in electrode material research. Ruiz-Hitzky et al. synthesized the graphene-like Sep nanocomposites for electrode materials of rechargeable lithium batteries [35], which showed better cyclability and Li-insertion properties than the nanostructured carbon without the silicate counterpart. Pan et al. used a simple and scalable process which manufactured a high-capacity [36], high-rate-performance, and low-cost sepiolite-sulfur cathode material, making it promising for the commercialization of lithium-sulfur batteries. Actually, antimony-doped tin oxide nanoparticles (Sb–SnO_2_) could be successfully coated on the surface of clay mineral (natural kaolinite, Kaol) to synthesize the kaolinite-based conductive material (Sb–SnO_2_)Kaol [37, 38], while Sep and kaolinite have similar physicochemical property. In this work, we reported a hydrothermal strategy to synthesize novel SnO_2_ **–**C@Sep nanocomposite. This kind of nanostructure could prevent the detachment and agglomeration of SnO_2_ to a certain extent and preserve the integrity during cycling, which could make it promising for comparison with high-cost and complex C-based materials.

## Methods

Sepiolite from sedimentary deposit in the central south of China was used in this study. All chemicals were analytical grade and used without further purification. Raw Sep was dipped in water and sieved to get rid of the coarse sand. Then sodium hexametaphosphate (0.8 wt%) was added into the pulp and dispersed with a high-shear dispersion homogenizer at 2000 rpm for 20 min, and the pulp was kept standing for 2 h. Finally, the suspension was filtered and dried to produce the final bunchy Sep.

Two grams of SnCl_4_·5H_2_O, 1.0 g sucrose, and 1.0 g Sep were dissolved in the mixture of 20 mL ethyl alcohol and 20 mL deionized water with ultrasonic for 30 min and stirring for 1 h; then the mixture was transferred into a Teflon-lined steel autoclave and statically heated at 200 °C for 12 h. The obtained brown precursor was filtered and washed several times with deionized water and alcohol. After drying at 60 °C overnight, it was calcined at 700 °C at a heating rate of 10 °C min^−1^ for 3 h in Ar atmosphere to prepare the final black product of the carbon- and SnO_2_-coated Sep (SnO_2_ **–**C@Sep). Similar procedure was employed for SnO_2_ and carbon-coated Sep (C@Sep).

X-ray diffraction (XRD) patterns of the samples were recorded on a DX-2700 X-ray diffractometer with Cu Kα radiation (*λ* = 0.15406 nm) at a scan rate of 0.02° s^−1^ and at 40 kV and 40 mA. Fourier transform infrared spectroscopy (FTIR) spectra of the samples were obtained between 4000 and 500 cm^−1^ on a Nicolet Nexus 670 FTIR spectrophotometer using KBr pellets. The morphology of the samples was observed by a JEOL JSM-6360LV scanning electron microscopy (SEM) with an accelerating voltage of 10.00 kV and JEOL JEM-2100F transmission electron microscope (TEM) operating at 200.00 kV.

The active materials were mixed with conductive carbon black (Super P) and binder (polyvinylidene fluoride) at a mass ratio of 8:1:1; an N-methyl pyrrolidinone (NMP) was added to form the electrode slurry, which was then coated on a copper foil to form the working electrode. The electrode was dried in vacuum at 120 °C for 12 h. The 2016-type stainless steel coin cells were assembled in a re-circulating argon glove box (Mikrouna Co., [H_2_O] < 1 ppm, [O_2_] < 1 ppm). The pure lithium foil (1 mm thick) was used as the counter electrode, and a Celgard 2400 membrane was used as a separator. The electrolyte consisted of a solution of LiPF_6_ (1 M) in ethylene carbonate and dimethyl carbonate (EC + DMC) (1:1 in volume). The cells were galvanostatic discharged and charged over the potential range from 0.01 to 3.00 V vs Li/Li^+^ at room temperature and current density (CD) of 0.1 mA cm^−2^ for cycle tests and 0.1~0.8 mA cm^−2^ for rate capability tests using a NEWARE battery test system. Cyclic voltammetry (CV) was implemented on a CHI660A electrochemical workstation at a scan rate of 0.1 mV s^−1^ between 0.0 and 2.0 V. Electrochemical impedance spectrum (EIS) measurements were performed using a CHI660A electrochemical workstation in the frequency range from 100 KHz to 0.01 Hz with an ac perturbation of 5 mV s^−1^. Coulombic efficiency (CE) indicates the rate between discharge capacity and charge capacity, CE = discharge capacity/charge capacity. CD is calculated by CD = *I*/*M* or *I*/*A*, *I* is electric current, *M* is the corresponding sample mass, and *A* is the cross-sectional area of the electrode.

## Results and Discussion

**Fig. 1:**
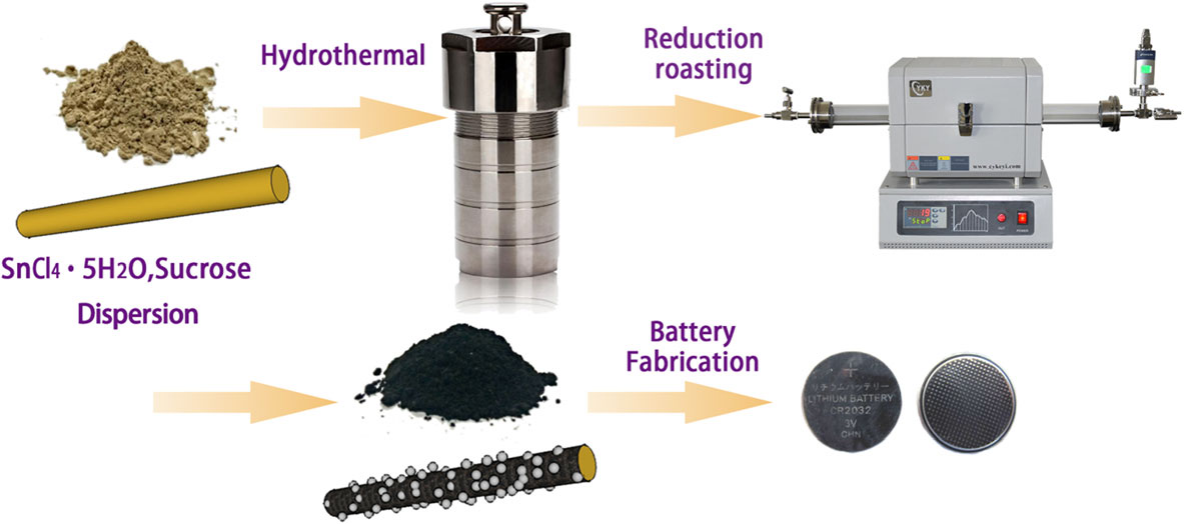
Schematic of synthesis of the SnO_2_ **–**C@Sep composites

**Fig. 2:**
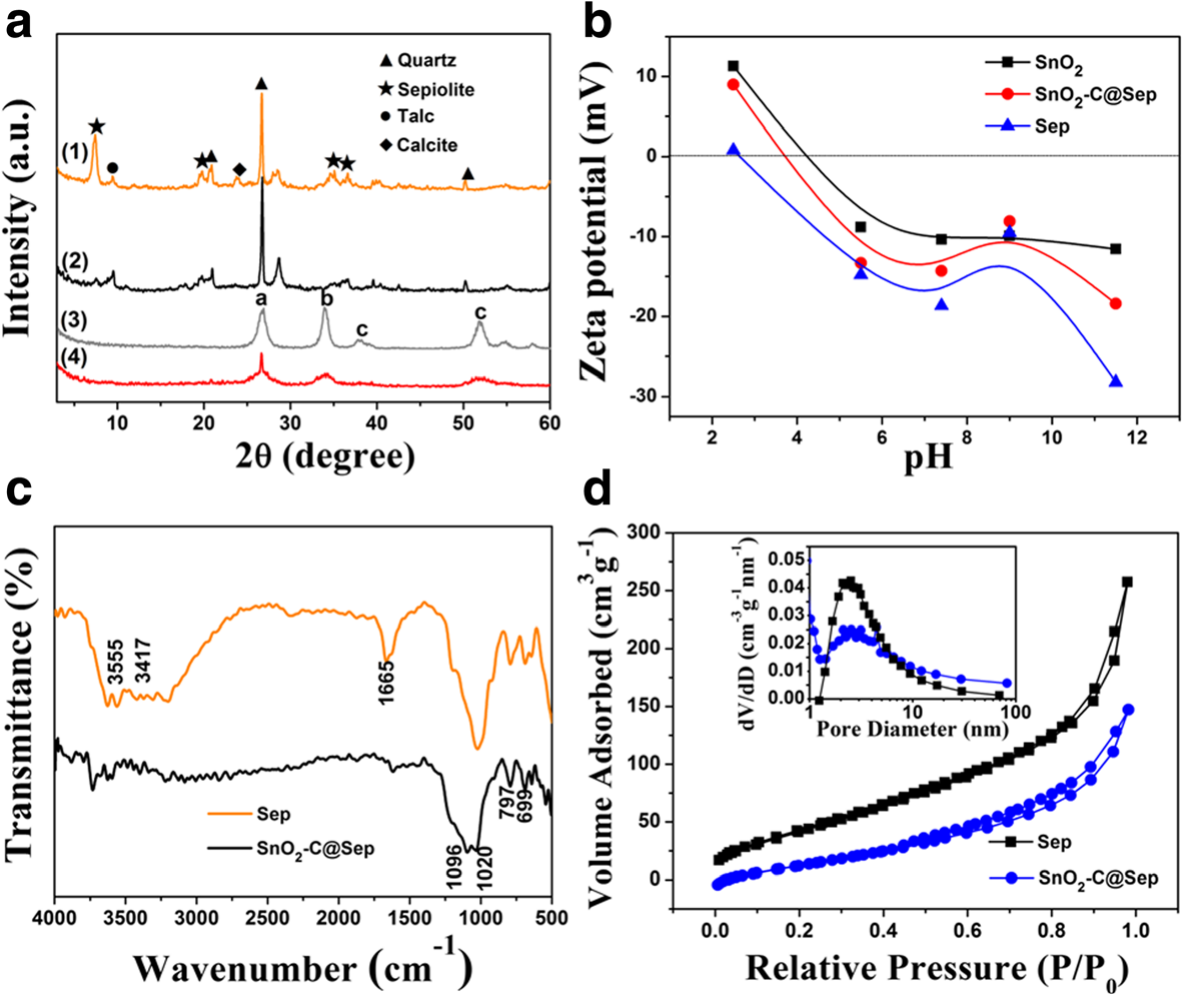
**A** XRD patterns of (*a*) pristine Sep, (*b*) C@Sep, (*c*) SnO_2_, and (*d*) SnO_2_–C@Sep. **B** Zeta potential of SnO_2_, SnO_2_–C@Sep, and Sep. **C** FTIR patterns of Sep and SnO_2_–C@Sep nanocomposites. **D** N_2_ adsorption-desorption isotherms of Sep and SnO_2_–C@Sep; the *inset* shows pore diameter distributions obtained by analysis of N_2_ adsorption

**Fig. 3:**
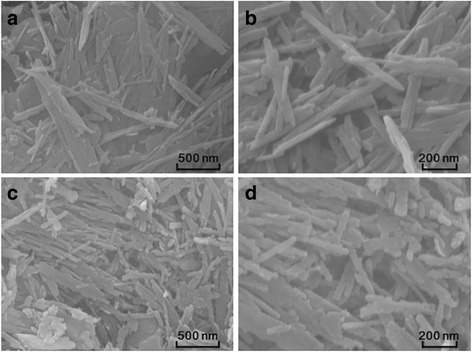
SEM images of **a**, **b** raw sepiolite and **c**, **d** SnO_2_–C@Sep

**Fig. 4:**
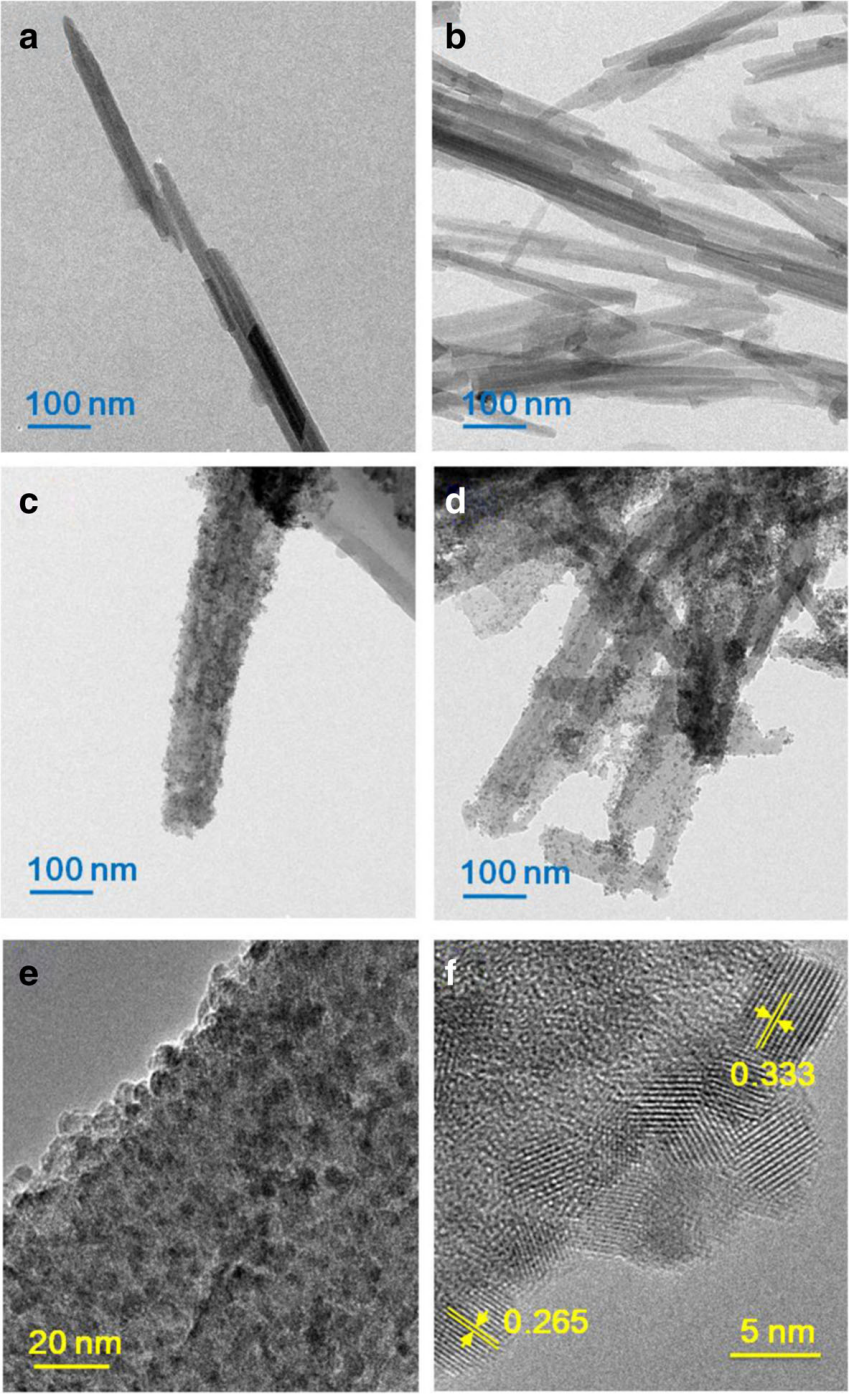
TEM images of **a**, **b** raw sepiolite and **c**, **d** SnO_2_–C@Sep. **e**, **f** High-resolution TEM (HRTEM) images of SnO_2_–C@Sep

Carbon particles are coated, and SnO_2_ nanoparticles are anchored on the surface of Sep together by a simple hydrothermal method (Fig. 1). Glucose in solution gradually accumulates on the surface of Sep at 200 °C for 12 h; hence, carbon microspheres decrease, and carbon nanoparticles on Sep increase [39]. For the typical SnO_2_ synthesis procedure, auxiliaries were added to prevent the effect of impurity [40, 41], no other reagents were added, and this hydrothermal condition could successfully realize the synthesis of SnO_2_ nanoparticles, both based on pertinent literature [42] and Figs. 2, 3, and 4. Sucrose will be transformed into amorphous carbonaceous material at 700 °C and Sep into anhydrous and partially as magnesium-dehydroxylated silicate [43].

Peaks of C@Sep composite obviously decreased compared with pristine Sep (Fig. 2A), which could verify the compact decoration of carbon on the Sep surface. Additionally, the broad peak of the SnO_2_ crystal observed at 2*θ* of 26.6°, 33.9°, 38°, and 51.8° closely matched with the (110), (101), (200), and (200), respectively [37, 38], according to JCPDS (No. 41-1445). The peaks of Sep in SnO_2_ **–**C@Sep composite disappeared while an overlap peak of quartz and SnO_2_ was found. The zeta potential of the Sep particles as a function of pH was tested at constant ionic strength of 0.1 M KCl and in 0.05% solid concentrations (Fig. 2B). The increase of the suspension pH results in an increase in the negative charge of Sep, which can be ascribed to either the adsorption of OH^−^ ions onto the positive charge center of Sep or the deprotonation of surface hydroxyl groups. The reaction of OH^−^ with dissolved cations to form metal hydroxides may also lead to the decrease of pH. As for Sep, SnO_2_ **–**C@Sep, and SnO_2_, all three lines decrease with the increase of pH, while IEP (pH_IEP_) values increase, which successfully verifies the composite of the material.

Figure 2C displays the FTIR absorption spectra of the characteristic bands for Sep and SnO_2_ **–**C@Sep samples. The multi-bands above 3000 cm^−1^ were mainly assigned to the stretching vibration of hydroxyl, adsorbed, and crystal water, and obvious OH bending vibration was observed at 1665 cm^−1^ [44]. The peaks of SnO_2_ **–**C@Sep composites became weakened and even disappeared, which may be related to the calcination dehydration of the composites. The band at 1020 cm^−1^ indicated the stretching vibration of O–Si–O of silicon-oxygen tetrahedron in Sep [45–47]. There appeared a new peak at 1096 cm^−1^ in SnO_2_ **–**C@Sep composites corresponding to the SnO_2_ addition.

The porosity of the materials was evaluated by using nitrogen adsorption-desorption isotherms measured at 77 K (Fig. 2D); the isotherms show a continuous uptake at low relative pressures. The increase in the nitrogen adsorption at a high relative pressure (*P*/*P*_0_ > 0.9) may arise from the interparticulate porosity associated with the macroporous structure of the samples. Hysteresis is apparently observed for Sep and SnO_2_ **–**C@Sep at a higher relative pressure range, which might be associated with the narrow crack aperture. The calculated BET surface area for Sep and SnO_2_ **–**C@Sep are 184 and 124 m^2^ g^−1^, respectively. In addition, SnO_2_ **–**C@Sep exhibits an isotherm of type IV, which is the characteristic isotherm of mesoporous structure [40]. The pore size distribution (PSD) curves (the inset of Fig. 2D) further suggest that Sep has a narrow pore size distribution with the pore width centered in the range of 2~3 nm, while that of SnO_2_ **–**C@Sep is about 2~4 nm. It is obviously observed that the pore size of the SnO_2_ **–**C@Sep is smaller than that of Sep. It manifests that the introduction of the SnO_2_ and carbon into Sep could block the pore of Sep, resulting in the decrease of surface area. The porous structure provides a short diffusion length for Li^+^ ions and electron during the electrochemical reaction. It is expected that such a rational design of SnO_2_ **–**C@Sep effectively integrates the intriguing functionalities of the three building blocks: the high electrical conductivity of C, the high theoretical capacity of SnO_2_, and the excellent structural stability of Sep, so it will be capable of greatly improving the practical usage of SnO_2_ for LIBs [48, 49].

SEM observations indicate that Sep was a nanorod shape with smooth surfaces (Fig. 3a, b). However, SnO_2_ **–**C@Sep nanocomposites have rougher surfaces (Fig. 3c, d). TEM images also demonstrated the morphology of Sep (Fig. 4a, b). Figure 4c –e clearly revealed that SnO_2_ and carbon nanoparticles were supported by sepiolite in the SnO_2_ **–**C@Sep nanocomposite with a diameter of ~5 nm. Besides, HRTEM image revealed an interplanar spacing of 2.65 and 3.34 Å (Fig. 4f), corresponding to the (101) and (110) lattice plane of the SnO_2_, respectively [50, 51].

**Fig. 5:**
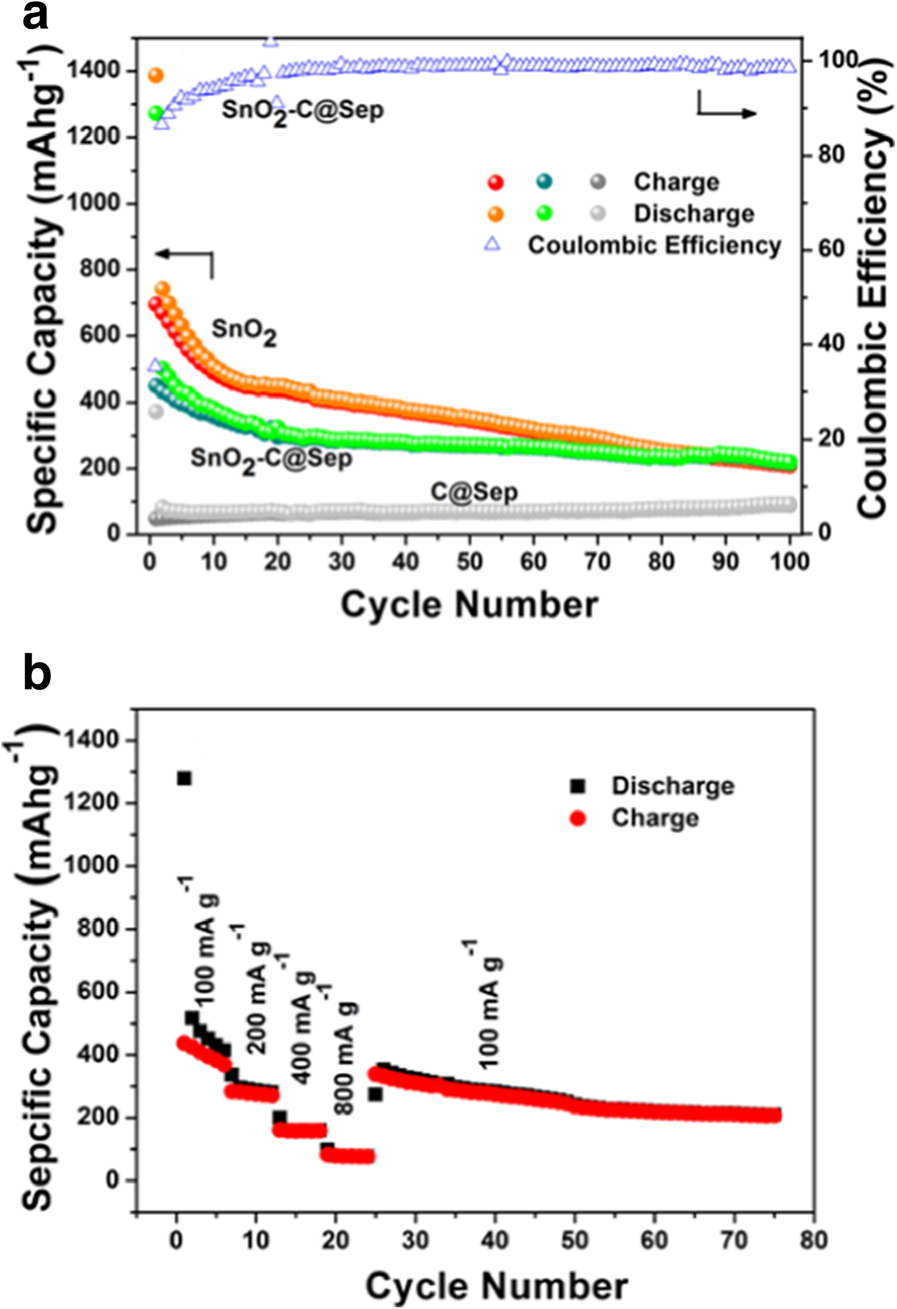
**a** Cycling performances of the SnO_2_–C@Sep nanocomposite, C@Sep, and SnO_2_. **b** Rate capabilities of SnO_2_–C@Sep nanocomposite

It is well known that there is a direct relationship between the electrochemical performance and the material nanostructures. Herein, reported SnO_2_ [17], experimental SnO_2_ **–**C@Sep, and experimental SnO_2_ were compared (Table 1). The results of the experiment show that discharge capacities (discharge electric quantity of active materials per unit mass) of the experimental SnO_2_ **–**C@Sep are more than those of the reported SnO_2_ after 50 cycle times and the discharge capacities of the experimental SnO_2_ **–**C@Sep are slightly more than those of the experimental SnO_2_ after 100 cycle times. The results of discharge capacities show better performance after long cycle times. As for the abovementioned materials, the large irreversible capacity (IRC) loss between the first discharge and the first charge is mainly attributed to the formation of Li_2_O and solid electrolyte interphase (SEI). The evidence for the formation of Li_2_O (SnO_2_ + 4Li^+^ + 4e^−^ → Sn + 2Li_2_O) and SEI is obtained from the cyclic voltammetry. Compared with SnO_2_ **–**C@Sep, experimental SnO_2_ (Fig. 5a), and reported commercial SnO_2_, the electrochemical performance of SnO_2_ **–**C@Sep as anodes of LIBs showed better cyclability and improved CE. The lower initial cycle test of SnO_2_ **–**C@Sep may be attributed to the existence of sepiolite and carbon. However, after 50 cycles, SnO_2_ **–**C@Sep shows higher discharge capacities than commercial SnO_2_, and after 100 cycles, SnO_2_ **–**C@Sep is superior to the experimental SnO_2_ in discharge capacity. Moreover, the cycling performances of C@Sep and its rate capabilities confirmed its improved stable cyclability and acted as a steady skeleton (Fig. 5a, b). SnO_2_/C fibers were introduced by electrospinning method [17]. By preparation, SnO_2_ **–**C@Sep has a simple one-step hydrothermal method. Electrochemical characterization by galvanostatic charge-discharge tests shows that the NF anodes have first discharge capacities of 1375.5 mAh g^−1^ at CD of 80 mA g^−1^. As for SnO_2_ **–**C@Sep, the first discharge capacity is 1271.6 mAh g^−1^. Both fibers can provide enough space to buffer the volume changes during the lithium insertion and extraction reactions.

**Table 1:** The comparison of discharge capacities for different samples

Sample	1st	2nd	10th	20th	50th	100th
Reported SnO_2_ [17]	1600.3	1100.1	646.9	485.9	145.1	–
Experimental SnO_2_	1383.9	741.6	507.3	448.5	353.1	213.0
Experimental SnO_2_ **–**C@Sep	1271.6	500.4	381.0	325.1	271.9	219.3

**Fig. 6:**
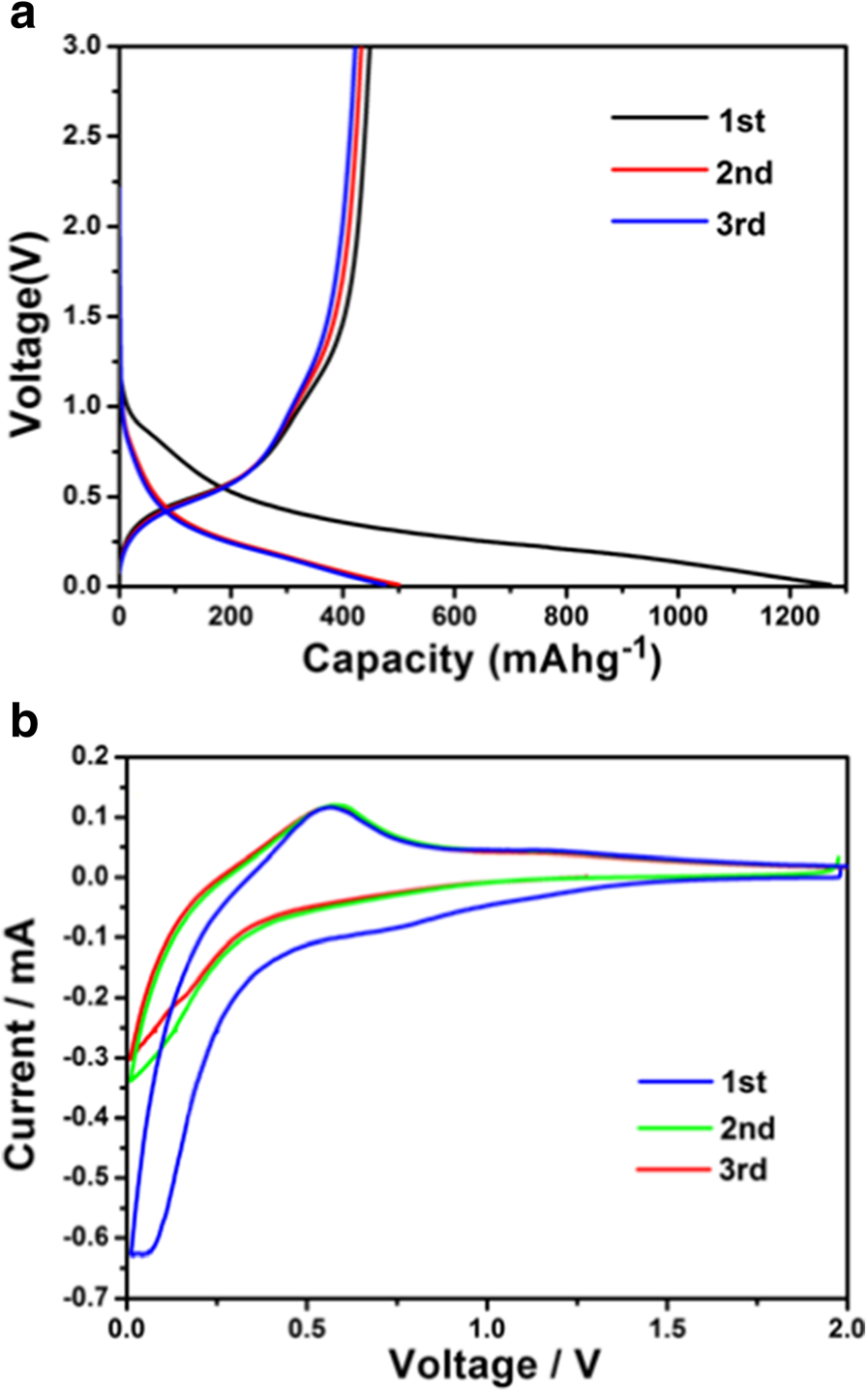
**a** Charge-discharge curves of the SnO_2_–C@Sep nanocomposite at a constant current density of 100 mA g^−1^ between 0.01 and 3 V. **b** Cyclic voltammetry curves of the SnO_2_–C@Sep nanocomposite for the first three cycles

The galvanostatic discharge/charge profiles for the first, second, and third cycles are presented with CD at 100 mA g^−1^ between 0.01 and 3 V (Fig. 6a). The initial plateau in the potential range from 0.9 to 0.6 V led to the formation of Li_2_O and Sn in the first discharge step, which corresponded to the reduction peak around 0.8 V in CV curves (Fig. 6b) with irreversible conversion reaction in which a SEI forms and it disappeared from the second cycle. The following long sloping discharge curve down to the cut-off voltage of 0.15 V indicated the alloying reaction and Li^+^ intercalation into the C@Sep. In the CV curves, the curves in the second and third circles at 0.2/0.5 V may represent the reaction of alloying reaction (Sn + xLi^+^ + xe^−^ ↔ Li_x_Sn, 0 ≤ *x* ≤ 4.4). Both discharge and CV curves after the first cycle almost kept overlapping, further demonstrating its good cycling stability.

**Fig. 7:**
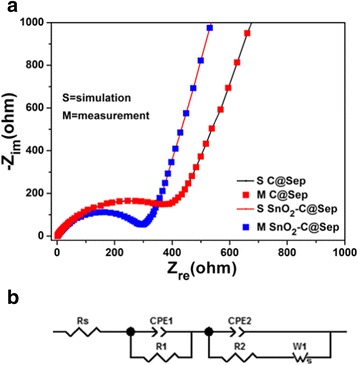
**a** Measured points and calculated lines for the impedance of C@Sep and SnO_2_–C@Sep nanocomposite anode. **b** The equivalent circuit used for the analysis of the impedance plots

Figure 7 shows the electrochemical impedance spectrum (EIS) tests of SnO_2_ **–**C@Sep lithium before charge and discharge. The depressed semicircles represent the high-frequency range and an angled straight line in the low-frequency range. Rs, R1, R2, and W1 are denoted as solution resistance, SEI film resistance, electrochemical reaction resistance, and Warburg impedance of the diffusion, respectively. The constant phase element (CPE) is defined as {Y(jw)}^−1^ replacing the capacitor element. Figure 7b is a nonlinear, least-square fitting calculation by using the equivalent circuit. The semicircle of SnO_2_ **–**C@Sep is smaller than that of C@Sep, suggesting the lower resistance of SnO_2_ **–**C@Sep and signifying the enhanced interparticle contact and improved conductivity [52]. Though the resistance of SnO_2_ **–**C@Sep is inferior to pure SnO_2_, it still reveals the improved lithium-ion storage properties compared to SnO_2_.

**Fig. 8:**
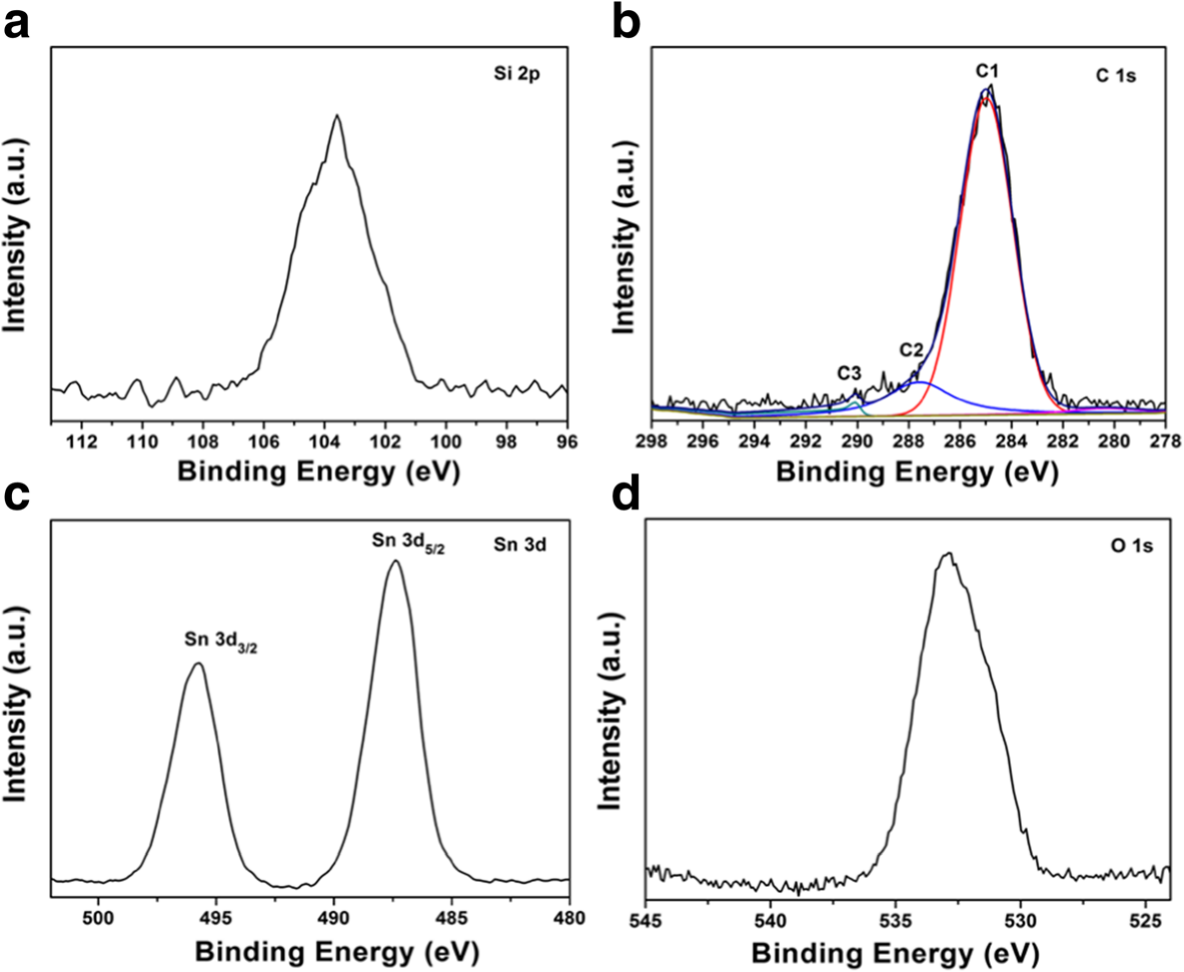
High-resolution XPS spectra of **a** Si 2p, **b** C 1s, **c** Sn 3d, and **d** O 1s of SnO_2_–C@Sep nanocomposite

SnO_2_ **–**C@Sep involved C 17%, Sn 33.21%, and Si 13.42% according to the elemental analysis and ICP-OES, so the content of active material reached about 59%, including 42.16% of SnO_2_. The peak at 103.6 eV is attributed to SiO_2_ (Fig. 8a), which is the main structure of sepiolite. The C1s spectrum of the sample can be separated into three peaks (Fig. 8b). The main peak at a binding energy of 285.3 eV is attributed to the C–O or C–C bonding, the peak at 287.6 eV is related to the carbonylate C (HO–C=O), and the peak at 289.0 eV corresponded to O–C=O components. As for the high-resolution Sn 3d spectrum (Fig. 8c), the two peaks at 495.8 and 487.4 eV are associated with Sn 3d 3/2 and 3d 5/2 orbitals, respectively, demonstrating that Sn atoms exist in the form of SnO_2_ and that the self-assembly based on the Van der Waals interactions does not alter their chemical nature. The O1s binding energy is 532.8 eV, suggesting that the oxygen atoms exist as O^2−^ species in the hybrid composites (Fig. 8d).

**Fig. 9:**
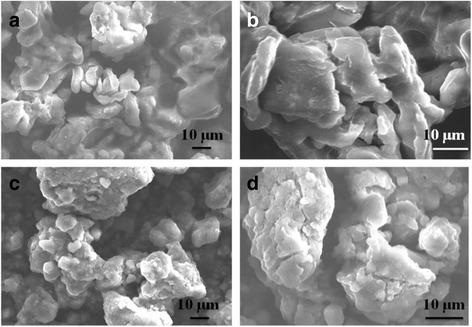
SEM images of **a**, **b** SnO_2_ and **c**, **d** SnO_2_–C@Sep extracted from cycled electrodes (100 cycles at 100 mA g^−1^)

To corroborate the volume expansion and pulverization of SnO_2_ and SnO_2_ on the fibroid sepiolite coated with carbon, morphological characterization was performed for a representative sample. We decomposed two cells (using SnO_2_ and SnO_2_ **–**C@Sep as anode materials, and cycled 100 cycles) after a certain number of cycles at CD of 100 mA g^−1^. The volume of SnO_2_ **–**C@Sep is much bigger than that of SnO_2_ (Fig. 9), and the cracking status is more significant, implying the better structural stability of the as-prepared composite during repeated discharge/charge cycling.

**Fig. 10:**
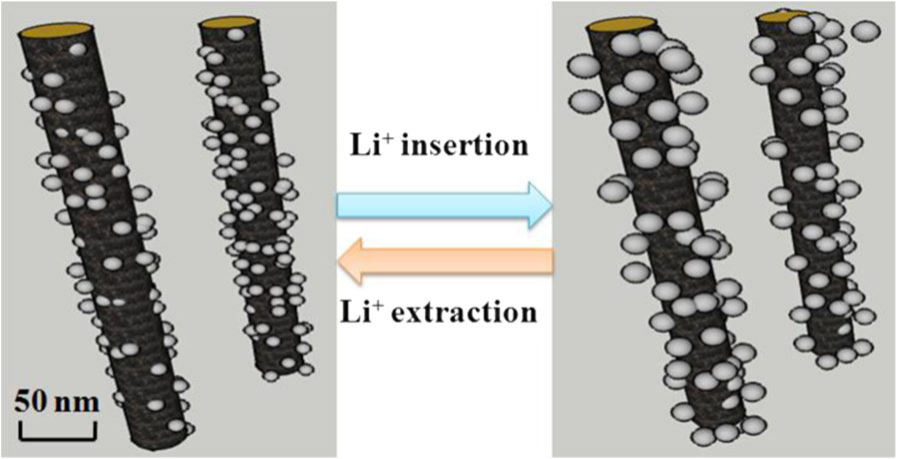
Schematic diagram of Li^+^ insertion/extraction from SnO_2_–C@Sep nanocomposite

The possible schematic diagram was shown in Fig. 10. The C@Sep nanocomposite was quite steady as shown in its cycling performance curve and contributing about 70 mAh g^−1^ discharge capacities only (Fig. 5a). The main insertion/extraction of Li^+^ can be intercalated into SnO_2_, also leading to the volume expansion and pulverization of SnO_2_. However, with the hybridization of Sep fibers, SnO_2_ nanoparticles were firmly anchored onto the surface of sepiolite. There are three possible reasons: firstly, in order to prevent aggregation and pulverization of SnO_2_, the Sep nanofibers show like fences. Elastic Sep framework can hinder the aggregation of SnO_2_ nanoparticles and provide enough space to buffer the volume changes during the lithium insertion and extraction reactions in SnO_2_. Secondly, amorphous carbon on sepiolite surface could effectively overcome the problem of poor conductivity of natural clay. Amorphous carbon could also promote the electron transfer during the lithiation and delithiation process. So, the smaller size of SnO_2_ particles was beneficial to the improved cycle performance. SnO_2_ nanoparticles were protected by carbon-coated Sep; for some of SnO_2_ nanoparticles, they were also coated with carbon film, keeping the higher electrochemical activity.

## Conclusions

Novel SnO_2_ **–**C@Sep nanocomposites were successfully developed via a simple one-step hydrothermal method. The sepiolite acted as a steady skeleton, carbon coating principally led the sepiolite from the isolated to the electric state, and anchoring it mainly with SnO_2_ nanoparticles makes it function. The SnO_2_ **–**C@Sep nanocomposite exhibits improved lithium storage properties with stable capacity retention and low cost, and the manufacturing process is simple and scalable. This may open up a new way to synthesize a variety of functional hybrid materials based on cheap and abundant clay and commercialization of lithium-metal oxide batteries.

## Abbreviations

CD: Current density
CE: Coulombic efficiency
CNTs: Carbon nanotubes
CPE: Constant phase element
CV: Cyclic voltammetry
DMC: Dimethyl carbonate
EC: Ethylene carbonate
EIS: Electrochemical impedance spectrum
FTIR: Fourier transform infrared spectroscopy
HRTEM: High-resolution TEM
ICE: Initial coulombic efficiency
IRC: Irreversible capacity
LIBs: Lithium-ion batteries
NMP: N-methyl pyrrolidinone
PSD: Pore size distribution
SEI: Solid electrolyte interphase
SEM: Scanning electron microscopy
Sep: Sepiolite
SnO_2_: Tin dioxide
TEM: Transmission electron microscopy
XRD: X-ray diffraction
